# (Trans)forming fitness: Intersectionality as a framework for resistance and collective action

**DOI:** 10.3389/fspor.2023.944782

**Published:** 2023-07-26

**Authors:** Deniece Bell, Saidur Rahman, R. Rochon

**Affiliations:** ^1^ Indigeneity Diaspora Equity and Anti-Racism in Sport (IDEAS) Research Lab, Faculty of Kinesiology and Physical Education, University of Toronto, Toronto, ON, Canada; ^2^ Department of Sport Management, Florida State University, Tallahassee, FL, United States

**Keywords:** intersectionality, queer (LGBTQ), resistance, fitness, inclusivity, joy, coaching education

## Abstract

Fitness is a lifelong pursuit, yet many LGBTQ2S+ ^1^ individuals are averse to group fitness or experiences in big box gyms. Due to the COVID-19 pandemic, virtual fitness programs offered the potential to facilitate opportunities for the greater inclusion of such individuals and the chance to connect, collaborate and advocate for a change in who and what defines fitness. Justice Roe, owner of Fit4AllBodies, utilizes the term ***fitness industrial complex*** to provide a framework to discuss the problems of exclusion. His explanation supports research documenting that bodies that are not “the norm”, defined by ableism, classism, (hetero)patriarchy and racism, fueled by white supremacy, are oftentimes viewed as “less than” in the fitness and recreation world ( 1– 3). Applying an intersectional framework, this article explores the possibilities for transformative collective action in fitness communities that removes barriers and challenges the injustices that contribute to racialized LGBTQ2S+ individuals feeling unwelcome. With the need to shift to virtual training spaces as a result of a global pandemic, and the rise in the public discourse surrounding racial injustices both on and offline, a sense of belonging and community is important, especially among groups that often face exclusionary practices, such as racialized LGBTQ2S+ community members. These individuals are at greater risk of losing opportunities to access fitness programs that can provide immense health and psychological benefits. What could an intersectional perspective on resistance in sport look like? Using the example of LGBTQ2S+ access to online fitness spaces during the prolonged global COVID-19 pandemic starting in 2020, we suggest that explicit coaching education and intentional communities, centered around social justice, are needed to address the historical roots of systemic oppression, accessibility, and social constructs tied to fitness.

*“Dismantling oppression and our role in it demands that we explore where we have been complicit in the system of body terrorism while employing the same compassion we needed to explore our complicity in our internalized body shame.”*

Sonja Renee Taylor

## Introduction

Body fitness is an integral part of health culture in the contemporary world. While the World Health Organization (4) cites immense health and psychological benefits from physical activity, they also state that globally, one in four adults, and over 80% of adolescents do not engage in adequate amounts of physical activity. For those that do, we see individuals of different age, gender, class, race and ethnicity accessing “health clubs, YMCAs, and recreation centers (5), also sometime referred to as gyms^2^, to maintain their health and fitness, and possibly achieve ‘normative’ Eurocentric fitness standards (6–8). Fitness can be defined in a number of ways, but in this instance, we cite popular workout techniques, such as aerobics and bodybuilding, which, in the 1980s and 1990s, saw a boom in fitness culture and the growth of commercial physical activity, measured by reps, sets, and pounds lost (9). Shaping and sculpting the body was encouraged by multiple sources, thus selling the idea that by engaging in fitness, one can become a “better” person, “taking care of God's gift … develop[ing] a healthy, religious, and morally righteous lifestyle”, an attractive thought to many individuals (10, p. 96). In this essay, physical activity taking place in recreational facilities, which we refer to as gyms and fitness spaces, are important for a variety of reasons. Gyms offer an opportunity to develop strength and endurance in one's body, feel a sense of empowerment and discipline in achieving an ideal body-type, progress gains in physical and mental strength, show a reduction of health risks, and an improvement in appearance (5, 11). As a result of aerobic and strength training done in fitness facilities, there are many psychological improvements, such as brain stimulation, assisting with cognitive abilities, and helping with dementia (12–14). In addition to reducing the risk of developing depression, studies have linked exercise to decreasing the levels of anxiety and depression in those with moderate forms of the illness (15). Resistance training can also support social and emotional gains including raising confidence, positive self-esteem, brightening mood, fighting depression, and improving sleep (16, 17). Engaging in physical activity can be seen as essential to one's overall health, as long as the social environment is enriching, and not exclusionary (18, 19).

Many people's association with fitness in later life can be tied to their experiences of sport and physical education in their early years, and more specifically, data supported by research on queer youth and physical education, reveals that gym sports and locker-room spaces can be alienating (3, 20, 21). Gyms and fitness spaces can be spaces of exclusion, with structural barriers and informal, or unspoken, cultural norms such as gender binary and heteronormative views, discouraging, and/or preventing certain groups, such as folks in larger bodies, those with (dis)abilities, women, racialized, and LGBTQ2S+ individuals, from accessing fitness and health centers (22–24). Though gyms and recreation facilities can be a critical node of intersectional exclusion in which LGBTQ2S+ minorities are under-represented and under-served, we look to present three case studies of fitness and recreation spaces engaging in intersectional praxis (25). Intersectionality as critical praxis allows us to examine the ways in which these businesses apply intersectional frameworks to their way of serving fitness enthusiasts. By bringing critical inquiry and critical praxis together, Collins & Bilge (25) cite the combined effect as something greater than separate parts, thus potentially creating new knowledge and practices around “fit”. This intersectional praxis creates dialogue and action, allowing all bodies room to benefit from gym and fitness spaces.

With the closure of many fitness and health centers due to the COVID-19 pandemic, numerous fitness programs shifted to online platforms. Virtual programs provide the potential to increase accessibility for many LGBTQ2S+ individuals, in contrast to “big box” gyms, also known as large corporate chain gyms in North America, such as Goodlife, LA Fitness, Planet Fitness, and Soulcycle, private gyms or even boutique style gyms. In this context, the pandemic represented an opportunity to make fitness spaces more inclusive and accessible to the LGBTQ2S+ community by eliminating barriers, so that they can reap the physical and psychological benefits of physical activity (19, 23, 24). Our writing investigates how transformational collective action in fitness spaces can enable social justice. By beginning our inquiry with the value of intersectionality as a framework to aid in identifying and removing barriers and challenges associated with racialized LGBTQ2S+ community's access and participation in gyms, we have built an account of how virtual platforms can assist in transforming gyms into spaces of resistance, accessibility and movement for joy.

### Writing on privilege and oppression

This essay builds on the authors' positionality as racialized and/or LGBTQ2S+ individuals who experience privilege and oppression in particular ways, including in sport and physical activity. Deniece, the first author, born in Ontario, Canada, identifies herself as a queer, Black individual with cultural roots coming from Guyanese and Jamaican parents. Her cultural background as a racialized human being provides her a unique perspective to navigate the world. She is able-bodied, and was privileged to access an athletic scholarship in the United States to pursue post-secondary education. Simultaneously, as a teacher, coach and trainer in higher educational institutions, and health and wellness spaces, she believes that self-actualization must correspond to social change and therefore, she tries to provide individuals with space to feel included and heard.

Saidur, the second author, identifies himself as a cisgender racialized male, with cultural roots in Bangladesh. Currently, he resides in Ontario, Canada, and pursues his doctoral studies in the Faculty of Kinesiology and Physical Education at University of Toronto. His research work focuses on decolonizing sport and physical activity with an aim to address injustice and oppression stemming from colonial and imperial values occurring in contemporary sporting spaces. Through his research, he intends to build an equitable and informed society where ethics and social justice will guide us into a harmonious future, dismantle forces of injustice and oppression, and ensure that every individual can exercise their right to self-determination.

The third author, Roc Rochon is a contributing author and cultural worker who is a Black, queer, trans nonbinary person. Roc is the founder of Rooted Resistance, a grassroots practice committed to reimagining bodywork for queer, transgender, and nonbinary people in the U.S. South. Movement outdoors is their form of refusal to commercialized notions of the body and an imperative place for a growing relationship with our bodies, each other, and the land. Roc is currently a doctoral candidate in the Department on Sport Management at Florida State University in Tallahassee, Florida (traditional and ancestral territory of the Apalachee Nation, the Muscogee Creek Nation, the Miccosukee Tribe of Florida, and the Seminole Tribe of Florida) with a focus on physical cultural studies and bodywork. Roc's studies are concerned with unsettling “sport” as a politicized cultural form through understanding how histories of land, power, subjugation, and colonialism interact with bodies (human and non-human). Roc's interest is in narrative stories and the ways that Black queer, trans, and nonbinary folk construct sporting counter-spaces that tend to collective Black life.

We combine these three perspectives to build an “outsider within” (26) account of the potential for gyms to become spaces of joy, liberation and self-actualization. Central to Black feminist activism is that liberation is not only for Black women, but for all; humanism shines a light on the fact that until all folks are free, none of us are free (26, 27). By creating space for all bodies, fitness culture has the potential to uplift those that are often subject to exclusionary practices.

### Fitness culture

What might have begun as an industry meant to promote healthy living, mainstream fitness sites have become a toxic environment filled with racism, misogyny, misogynoir, anti-trans bigotry and similar intolerances to gender non-binary individuals, making fitness spaces unsafe or unwelcoming for people in all their sexual and gender diversity (5, 28–31). Advertising and media have also transformed fitness into a site for the commodification of the body, which reproduces the hegemonic middle-class whiteness that is normalized by neoliberal commercial culture (32, 33). Narratives of idealized bodies as having a certain size to be fit, produced by globalized media, impact views about fitness and health that “other” those who are not considered to be the norm (8, 9, 34). Fitness culture is ever-evolving, but has had some consistent norms.

Overall, the fitness culture can be conceptualized in several ways. First, Glassner (35) describes fitness culture as a neoliberal market-led, demand-driven phenomenon of body transformation, in which a preoccupation with perfectly fit bodies exists, and is only afforded to the bodies that conform to hegemonic cultural expectations of perfection. In an effort to achieve the fit body, exercisers aim to become a copy of the bodies they see, rather than the images/media being a representation of the real (35, 36). Secondly, Maguire (5, 37) comments that the culture of fitness goods and services has shifted from social reform, to self-reform. Fitness cultures are explained as a supply-driven phenomenon of commercialization and commodification, in which “being fit” is about social capital for success in neoliberal society, and possessing the resources to undertake the “project of the self” in a competent fashion. This means minimizing health risks to increase productivity and maximizing the market value of your body (5). Fitness, then, is a measure of aptitude for life in consumer culture and a service economy (5). A third account, offered by Sassatelli (38), is of fitness cultures as a place of consumption, fueled by both the consumers and producers, where individual lives, identities and bodies become both the product and producer, that documents the lived experiences of gym goers' fun and frustration, shown through ethnographic methods (38). Fourth, and critical to our purposes, Justice Roe Williams, owner of Fitness 4AllBodies, discusses the term ***fitness industrial complex*** to describe fitness culture: the transformation of fitness into an industry that denigrates bodies that do not always conform to dominant narrative surrounding what defines “fit”; a culture based on capitalism, whiteness, and masculine heteronormative body standards. Roe's notion of the fitness industrial complex offers a useful framework to discuss the problems of exclusion. As Roe, from Fitness for All Bodies (2), explains, there is a need for fitness professionals to consider how “our bodies are connected to systems of oppression, how those systems are reinforced by the fitness industry, how to develop a social justice lens and importantly, how to apply this knowledge to their work with all populations” (2). The demand and supply logic of fitness is instrumental for the development of the Fitness Industrial Complex, while at the same time, the dialectics of the fitness culture offers a way of looking at the privilege and oppression that individuals encounter because of how the gym is shaped by dominant systems of difference-making, such as gender, class, race and sexuality.

The Fitness Industrial Complex defines and maintains power over our bodies through the lens of privilege. Dominant representations of fitness and gym culture teach users what it means to be fit and well in their bodies. We suggest, however, that this often ignores the complex aspects of race, gender, class, identity, ability and body shape, pointing to the value of bringing an intersectional perspective to an analysis of gym use and fitness culture.

### Intersectionality

Founding members of the Combahee River Collective (CRC) were Black feminist/womanist lesbian social activists. In a statement from the CRC (39), they centered their lived racialized and gendered experiences in a way that was intersectional. In their statement they discuss the inseparability of their stories stating that, “our own oppression is embodied in the concept of identity politics. We believe that the most profound and potentially most radical politics come directly out of our own identity, as opposed to working to end somebody else's oppression” (p. 4). To honor their full lived experience, the Combahee women understood that a separatist politics regarding race, gender, sexuality, and class was antithetical to their (or anyone else's) liberation. Thus, the idea of intersectionality is not new; however, the term coined by Kimberle Crenshaw in 1989 provides a framework that can be used to support or create best practices within health and fitness. While there is no consensus on the definition or description of an intersectional methodology, Watson (40), explains it as “acknowledging and accounting for the consequences of difference (p. 315)”, making space to acknowledge systems that are in place to continuously benefit some, at the expense of others. In their research in leisure (and sport) studies, Watson (40) uses intersectionality as a methodological tool to contextualize the differences among individuals, instead of categorizing the various contexts of their life, which cannot be extrapolated from who they are. McDonald (41) suggests the use of intersectionality to understand the “complicated character of whiteness” (p. 152) and how its application can “reveal the tensions between experience, consciousness and sport” (p. 154). It is clear that among researchers there are various uses, and needs, for the application of an intersectional framework.

In relationship to the fitness industrial complex, intersectionality can have a positive role in moving forward resistance and collective action in sport by lifting up the voices typically found in the margins, and by supporting inclusive practices.

*“Intersectionality is defined as the interconnected nature of social categorizations such as race, class, and gender as they apply to a given individual or group, creating overlapping and interdependent systems of discrimination or disadvantage. They do not exist separately from each other but are interwoven and linked together. It is meant to articulate the overlapping systems of oppression that are faced by those who are in marginalized positions – either by social determinants of health, geography or facets of their identities” (* 42 *)*

From a Canadian sport perspective, safety, one of the eight principles explored in the 2020 Canadian Sport for Life Summit, was explained as a way to support success in sport from a participant/people-centered approach. They examined safety using an intersectional lens, without explicitly stating the term.

*“Safe would be lived by applying for funding to create a safer physical space (ex. change rooms). Through opportunities to share our stories, and to examine the different needs of the people in the spaces and places so that we can better plan to support the many diverse people who will be part of our programs or who deserve to be included but haven*’*t been planned for properly yet” (*42*)*.

There are many opinions on the importance and/or difficulty of intersectionality as a theory (43), as a methodological tool, and as a framework. Yet, the multiplicity of binaries (black/white, male/female, heterosexual/homosexual) that operate within fitness spaces continues to be relevant to the gym experiences of many individuals. Continued research focusing on the intersections of race, class, gender, and sexual identity as it relates to sport demonstrates that an intersectional approach is necessary. In December 2020, the Canadian Television Network (CTV) reported that every study over the past 15 years has shown that LGBTQ2S+ youth play sports at a lower rate than “straight” kids. Thus, these youth, and certainly adults, are doubly impacted by discrimination, while losing out on the mental health benefits of physical activity and sport (21, 44). Additionally, within the LGBTQ2S+ community, the context of varying identities matter. Black/racialized LGBTQS+ community members experience barriers within fitness and wellness that are amplified by their location in interlocking systems of difference-making and oppression (3, 45, 46). How, in this context, can intersectionality as a theory be leveraged to create change?

### Transformative collective action in fitness communities: case studies

In what follows, we explore the notion that the social movement(s) and collaborations enacted *via* virtual spaces to resist the status quo could offer opportunities to create intersectionally inclusive fitness practices and communities. *If* they could succeed in providing inclusive and affirming content and environments, how might that allow for like-minded individuals to challenge dominant narratives, and most importantly, create a sense of community and belonging? It is from this sense of community that individuals and organizations can increase their capacity to extend their reach. To explore this possibility, we look to the experiences of Black and/or queer fitness professionals who responded to the COVID-19 pandemic by introducing virtual fitness programmes, education and training that encourage change through liberatory praxis.

The COVID-19 pandemic was characterized by widespread lockdowns that saw in-person fitness spaces close, forcing fitness providers to develop new delivery models that engaged fitness space users *via* online platforms. The three programmes elaborated on below, deconstructed the notion of “fitness” and used grassroots community-based curricula that shifted hegemonic ideology around bodywork practices. These Black and/or queer and trans practitioners are creating pathways and re/membrances of being in relationship with their bodies and with their clients as a liberatory experience.

We describe these three models as follows: (1) A ***size-inclusive approach***, in which Fraiser, of Lift of Strength and Wellness, focuses on education for coaches with an emphasis on acknowledging all aspects of an individual, both visible and invisible, that need to be accounted for in their fitness journey; (2) ***An Agency Based Approach***, in which Parker, of Decolonizing Fitness stresses lived experience as valued and central to one's movement journey, while also providing affirming resources and care for queer and/or racialized individuals; lastly, (3) ***A Social Justice Based Approach***, where the focus is on addressing the systemic and historical structures that have informed the development of a fitness culture that values “Othering” as a means to continue upholding notions of whiteness as the norm. Forged during the COVID-19 pandemic as a means of virtually engaging diverse communities in online fitness spaces, we suggest that these models offer opportunities for learning that will continue to be relevant to both online and in-person fitness delivery going forward.

### Lift off strength and wellness: a size-inclusive approach

Damali Fraiser, certified Kettlebell Instructor and Nutrition Coach, runs Lift Off Strength and Wellness, virtually, and onsite in Ontario, Canada. Her focus on size-inclusive coaching for kettlebells led to the creation and launch of Coaches Corner. In the Lift Off Strength and Wellness website, it is mentioned that “Coaches corner is an 8-week kettlebell teaching course where we, together, learn how to coach Kettlebell Athletes and adapt for any body shape, size or ability. We teach person-based coaching and creating safe spaces for very personal fitness journeys” (47). The first course was run in January 2021, in response to exclusionary fitness practices and expectations on bodies to fit a certain mold (read: thin, able bodied, and white). On this initiative, Damali stated, “I created this course to break down the barriers to kettlebell training, making it accessible to all bodies regardless of race, religion, gender, ethnicity, body size, ability or sexual orientation” (47). Further to this, Frasier has developed a KettleBell in Black instagram community focused on love, connection, and self-care for black women. Thus, this virtual initiative, through its acceptability and accessibility to all groups of people, aims to eradicate barriers for marginalized people by providing them a safe space for physical activity, fitness and wellness.

### Decolonizing fitness: an agency based approach

Ilya Parker shares knowledge on various platforms, and has continued expanding his own business, Decolonizing Fitness, where he states on his website that “Decolonizing Fitness is not a gym, but an incredible educational resource for coaches, trainers, studio owners, and anyone who is interested in unlearning toxic fitness culture” (48). Ilya defined toxic fitness culture as “Social characteristics, language and habits that promote/reinforce ableism, fatphobia, racism, classism, elitism, body shaming/policing, LGBTQIA+ hatred under the guise of fitness and wellness” (49). According to Parker (49), in the fitness culture, the dominant group, comprising able-bodied, toned, traditionally attractive, young, cisgender, heterosexual people act as the gatekeepers to define “ideal” ways of engagement and embodiment of fitness; whereas other groups of people with marginalized identities are excluded to assert agency over their bodies, access the fitness culture based on their actual needs and feel alienated in different fitness and wellness spaces. Applying a decolonial lens, Decolonizing Fitness aims to build accessible and supportive physical and virtual spaces for various groups of bodies, encourage movements that produce good feeling and agency about one's own body, respect the anti-racial movements led by Black, Indigenous and People of Color (BIPOC), and acknowledge one's own lived experience surrounding their bodies, even if it does not fit the “ideal” standard set by the dominant group.

### Fitness4AllBodies: A social justice based approach

Justice Roe Williams is a trans body positive activist who runs Fitness 4 All Bodies (F4AB). His course, titled “Deconstructing the Fitness Industrial Complex: Identifying Power Dynamics & Moving Toward Connection” is built to discuss and understand “ways we can reshape our practice and reframe the relationship that we have with fitness, movement, and bodies” (2). The six-week long course aims to unpack the Fitness Industrial Complex, identify the roots of toxic masculinity in fitness culture, comprehend sex and gender as social constructs and ensure accessibility for all bodies, identities, shapes and abilities by reframing language, and moving beyond inclusion (2). Fitness 4 All Bodies trains coaches, gym/studio owners, and people associated with the fitness industry, to educate them about the link between our bodies and systems of oppression, and the reinforcement of different body and racial stereotypes in the fitness industry. The goal is to enlighten and equip the participants with the notion of social justice, which they can apply in their jobs and places to challenge and dismantle “patriarchal, white supremacist bodily ideals” (50, p. 5). Thus, focusing on belongingness, past history, leadership development, education and healing, Fitness 4 All Bodies works toward transforming the fitness space to address structural injustice and facilitate vulnerable groups to engage in meaningful conversation, build community connection and liberate their bodies through inclusive physical activity.

## Conclusion

Blackness, maleness, sexual identity and other aspects of the self have been, and continue to be, explored in isolation. Intersectionality encourages a justice-oriented approach for society to recognize all parts of an individual; to see people as whole and complete beings. This belief aligns with the thinking of Audre Lorde (51), who wrote: “There is no such thing as a single-issue struggle because we do not live single-issue lives” (p. 138). We can view our lived experiences as knowledge (52–54). By being modern day griots, we – Queer, BIPOC must tell our unique stories, as well as share our collective experiences, while we constantly engage with, and survive structural and institutional repression. Further research needs to be done regarding the limited recreation and sport in a safe, inclusive environment *for* racialized LGBTQ2S+ individuals, and *with* racialized LGBTQ+ instructors and mentors. The COVID-19 pandemic saw the fitness industry shift from traditional gym spaces to online programming. As the case studies above illustrate, this also represented an opportunity to develop new communities of practice and uplift the voices that counter the dominant narrative of white supremacist cis-heteropatriarchy messaging in the fitness world. While these communities existed in different iterations before COVID-19, the implications of these case studies and the continuation of these online communities each continues today, hosting workshops, training, and creative content for continued learning. Additionally, the founders of two of the case studies—Fitness 4 All Bodies and Lift Off Strength and Wellness both facilitate courses (online) where practitioners can obtain continued education units (CEUs) or a certification on particular content, and both host virtual events. The insights from online engagement with such courses allow an opportunity for LGBTQ2S+ fitness trainers, coaches, and owners to actively be a part of experiencing what modeling the way toward what anti-racist and inclusive fitness training can resemble. Further, insights from what has grown online can now offer insights to the ways in which fitness instructors deliver inclusive programming in-person at gym spaces and during specific certification and workshop programs. Though no space can be replicated without the coaches and trainers doing the continued individual and collective work of unlearning dominant narratives about bodies, these case studies challenge the fitness industrial complex. Coaches and fitness spaces are challenged to live out intersectionality in their everyday practices.

Racialized and marginalized individuals are not the only people that have the ability to create spaces that are affirming and inclusive. Tate (55) comments on black communities suffering from research fatigue, therefore it is the collective responsibility of the sector and the industry to enable change. It begins by engaging in difficult conversations where those who partake, knowing and unknowingly, in toxic fitness culture develop an awareness of intersectionality and how it impacts physical, social and emotional health. Developing a common language allows for individuals to take stock of where they hold privilege and where they contribute to the oppression of others.

*If you're a woman, if you're a person of color, if you're gay, lesbian, bisexual, transgender, if you're a person of size, a person of intelligence, a person of integrity, then you're considered a minority in this world. And it's going to be really hard to find messages of self-love and support anywhere. It's all about how you have to look a certain way or else you're worthless. For us to have self-esteem is truly an act of revolution and our revolution is long overdue*.

- Margaret Cho (56)

Intersectionality, used as a framework, in conjunction with liberatory thinking provides an opportunity for the recognition of the multiplicities that exist within our world (43, 57). Understanding and applying intersectionality is not a solution, by any means, to breaking down the systemic barriers that exist. However, it is a step in recognizing inequitable patterns and systems and building alternative structures of practice that humanize the individuals that walk through fitness doors in all of their diversity.

## Data Availability

The original contributions presented in the study are included in the article/Supplementary Material, further inquiries can be directed to the corresponding author.

## Footnotes

^2^The Cambridge dictionary defines gym as a place or club where you can go to exercise using machines, weights, and other equipment, or an open space where a large room with equipment for exercising the body and increasing strength, or space for playing sports:
